# Impact of deep learning reconstruction on intracranial 1.5 T magnetic resonance angiography

**DOI:** 10.1007/s11604-021-01225-2

**Published:** 2021-12-01

**Authors:** Koichiro Yasaka, Hiroyuki Akai, Haruto Sugawara, Taku Tajima, Masaaki Akahane, Naoki Yoshioka, Hiroyuki Kabasawa, Rintaro Miyo, Kuni Ohtomo, Osamu Abe, Shigeru Kiryu

**Affiliations:** 1 Department of Radiology, The University of Tokyo Hospital, 7-3-1 Hongo, Bunkyo-ku, Tokyo, 113-8655 Japan; 2 Department of Radiology, International University of Health and Welfare Narita Hospital, 852 Hatakeda Narita, Chiba, 286-8520 Japan; 3 Department of Radiology, The Institute of Medical Science, The University of Tokyo, 4-6-1 Shirokanedai, Minato-ku, Tokyo, 108-8639 Japan; 4 Department of Radiology, International University of Health and Welfare Mita Hospital, 1-4-3 Mita, Minato-ku, Tokyo, 108-8329 Japan; 5 Department of Radiological Sciences, School of Health Sciences at Narita, International University of Health and Welfare, 4-3 Kozunomori, Chiba, 286-8686 Japan; 6 International University of Health and Welfare, 2600-1 kitakanamaru, Otawara, Tochigi 324-8501 Japan

**Keywords:** Artificial intelligence, Deep learning, Magnetic resonance angiography, Head

## Abstract

**Purpose:**

The purpose of this study was to evaluate whether deep learning reconstruction (DLR) improves the image quality of intracranial magnetic resonance angiography (MRA) at 1.5 T.

**Materials and methods:**

In this retrospective study, MRA images of 40 patients (21 males and 19 females; mean age, 65.8 ± 13.2 years) were reconstructed with and without the DLR technique (DLR image and non-DLR image, respectively). Quantitative image analysis was performed by placing regions of interest on the basilar artery and cerebrospinal fluid in the prepontine cistern. We calculated the signal-to-noise ratio (SNR) and contrast-to-noise ratio (CNR) for analyses of the basilar artery. Two experienced radiologists evaluated the depiction of structures (the right internal carotid artery, right ophthalmic artery, basilar artery, and right superior cerebellar artery), artifacts, subjective noise and overall image quality in a qualitative image analysis. Scores were compared in the quantitative and qualitative image analyses between the DLR and non-DLR images using Wilcoxon signed-rank tests.

**Results:**

The SNR and CNR for the basilar artery were significantly higher for the DLR images than for the non-DLR images (*p* < 0.001). Qualitative image analysis scores (*p* < 0.003 and *p* < 0.005 for readers 1 and 2, respectively), excluding those for artifacts (*p* = 0.072–0.565), were also significantly higher for the DLR images than for the non-DLR images.

**Conclusion:**

DLR enables the production of higher quality 1.5 T intracranial MRA images with improved visualization of arteries.

## Introduction

Defects in intracranial vessels cause several neurological diseases. Strokes are common and affect one in four people [1, 2]. They are the second-leading cause of death and the third-leading cause of disability in adults worldwide [1]. Most strokes are ischemic strokes [2]. According to the Stop Stroke Study-Trial of ORG 10,172 in Acute Stroke Treatment (SSS-TOAST), cases of acute ischemic stroke can be classified into the following predetermined etiologic categories: large artery atherosclerosis, small-artery occlusion, cardioaortic embolism, undetermined causes and other causes. Optimal use of the SSS-TOAST classification relies on imaging of the vessels and brain [3]. Subarachnoid hemorrhage accounts for 5–10% of all strokes in the United States [4]. Many subarachnoid hemorrhages without any preceding trauma are caused by the rupture of an intracranial aneurysm [5]. Magnetic resonance angiography (MRA) allows the evaluation of acute ischemic stroke with SSS-TOAST classification [3] and detection of intracranial aneurysms [6].

Time-of-flight MRA (hereafter, time-of-flight MRA is referred to as MRA unless otherwise specified) is the most common MRA imaging technique. MRA images are obtained using magnetic resonance imaging (MRI) units with static magnetic fields of 1.5 T, 3 T, etc. MRA images obtained with MRI units with higher static magnetic fields are associated with some advantages. These images have higher spatial resolution and less image noise, allowing clearer depictions of large and small intracranial arteries [7, 8]. However, these MRI units are associated with higher costs and are less accessible. It is also safer to perform MRI examinations using lower static magnetic fields for patients with metal implants. MRI units with a magnetic field of 1.5 T are widely available. However, as indicated above, MRA images obtained from the 1.5 T MRI units are associated with lower spatial resolution and greater image noise.

Recently, deep learning has gained increasing attention in the field of radiology [9–11]. Recent studies have revealed that deep learning image analysis allows lesion detection, staging and differential diagnosis with MRI [12–14] and computed tomography [15, 16]. However, the potential of deep learning is not limited to these tasks. In recent years, the application of deep learning to image processing has also been investigated [17]. Deep learning reconstruction (DLR), a new image reconstruction algorithm based on deep learning [18], is an example of this application that is now available from MRI vendors. This algorithm enables the reduction of image noise [18–20], which is one disadvantage of 1.5 T MRA.

This study aimed to evaluate whether DLR improves the quality of intracranial MRA images obtained using a 1.5 T MRI unit.

## Methods

This retrospective study was approved by our institutional review board (20-Nr-056). The requirement for written informed consent was waived.

### Patients

All the consecutive patients who underwent brain MRI examinations at a single institution between October 2020 and February 2021 [40 patients; 21 men and 19 women; mean age ± standard deviation (SD), 65.8 ± 13.2 years] were included in this study. Indications for brain MRI examination included the following: dizziness (*n* = 9), brain infarction (*n* = 8), cerebral aneurysm (*n* = 6), screening before cardiac surgery (*n* = 4), headache (*n* = 4), trigeminal neuralgia (*n* = 2), and others (*n* = 7).

### MRA imaging

Three-dimensional time-of-flight MRA imaging was performed using a 1.5 T MRI unit (Vantage Orian; Canon Medical Systems). The imaging parameters for obtaining the MRA images were as follows: repetition time, 21 ms; echo time, 6.8 ms; flip angle, 20 degrees; number of averages, 1; field of view, 200 × 200 mm; acquisition matrix, 384 × 208; pixel bandwidth, 122 Hz; pixel size, 0.2604 mm; slice thickness, 1.1 mm; slice interval, 0.55 mm; parallel reduction factor, 3; and receive coil, Atlas Head Neck (16 channel). MRA source images were reconstructed with and without the DLR technique (Advanced Intelligent Clear IQ Engine; Canon Medical Systems) (DLR images and non-DLR images, respectively) [18].

All the MRA source images were anonymized and exported from the picture archiving and communication system in Digital Imaging and Communications in Medicine format. Maximum intensity projection (MIP) images with coronal and axial views were generated using ImageJ software (https://imagej.nih.gov/ij/) from the source MRA images.

### Quantitative analyses

Quantitative analyses were performed using the source MRA images on ImageJ software. A radiologist (11 years of imaging experience) drew round regions of interest (ROIs) on the basilar artery and cerebrospinal fluid of the prepontine cistern (diameters of approximately 1.5 mm for all). The peripheral parts of the basilar artery were not included in the ROIs to avoid partial volume effects. The ROIs were copied and pasted to ensure that their location and size were identical on the DLR and non-DLR images. We recorded the average signal intensities associated with the basilar artery (SI_BA_) and cerebrospinal fluid (SI_CSF_). The SD of the signal intensity for cerebrospinal fluid (SD_CSF_) was also recorded. The signal-to-noise ratio (SNR) and contrast-to-noise ratio (CNR) for the basilar artery (SNR_BA_ and CNR_BA_, respectively) were calculated using the following formulae:

$$ {\text{SNR}}_{{{\text{BA}}}} {\text{ = SI}}_{{{\text{BA}}}} {\text{/SD}}_{{{\text{CSF}}}} $$

$$ {\text{CNR}}_{{{\text{BA}}}} = ({\text{SI}}_{{{\text{BA}}}} - {\text{SI}}_{{{\text{CSF}}}} )/{\text{SD}}_{{{\text{CSF}}}} $$

The radiologist also drew a line ROI with the length of around 10 mm passing the center of the basilar artery and obtained plot profile of SI along the line ROI. The ROIs were copied and pasted to ensure that their location and size were identical on the DLR and non-DLR images. From the plot profile of SI, full width at half maximum (FWHM) of the basilar artery was calculated.

### Qualitative analyses

Qualitative analyses were performed mainly using the source MRA images. Axial and coronal MIP images could be referenced to make the evaluation easier. The radiologist randomized all 80 image sets (40 patients with two image sets each). Two additional experienced radiologists (readers 1 and 2 with imaging experience of 18 and 7 years, respectively) performed the qualitative analyses. They were blinded to whether each image set was DLR or non-DLR. They independently evaluated single image set at a time. They evaluated the image sets in terms of the following: depiction of structures (supraclinoid segment of the right internal carotid artery, right ophthalmic artery, basilar artery and right superior cerebellar artery) (5 = sharp depiction, 4 = minimal blurriness or heterogeneity, 3 = identifiable for most parts with some parts unidentifiable or blurred, 2 = identifiable for only some parts and with most parts unidentifiable or blurred, and 1 = unidentifiable), subjective image noise (5 = minimal noise, 4 = less than standard noise, 3 = standard noise, 2 = more than standard noise, and 1 = unacceptable noise), artifacts (e.g., laminar-flow related and magnetic susceptibility) in the right internal carotid artery (3 = no artifacts, 2 = minimal artifacts, and 1 = unacceptable number of artifacts) and overall image quality (5 = excellent, 4 = better than standard, 3 = standard, 2 = less than standard, and 1 = unacceptable).

### Statistical analyses

We used EZR, a graphical interface of R, for most of the statistical analyses [21]. Additional analyses were performed based on kappa analysis in Python and scikit-learn (https://scikit-learn.org/stable/). The normality of the data distributions for the quantitative analyses was assessed using the Shapiro–Wilk test. Because all data did not follow normal distributions (*p* < 0.022), Wilcoxon’s signed rank tests were performed for the quantitative analysis. Wilcoxon signed-rank tests were also performed to compare the qualitative scores. A *p* value of < 0.05 was considered to indicate statistically significant differences. Cohen’s weighted kappa analysis (with quadratic weight) was performed to evaluate the interobserver agreement. The following values were used to indicate agreement: 0–0.20, poor; 0.21–0.40, fair; 0.41–0.60, moderate; 0.61–0.80, good; and 0.81–1.00, excellent.

## Results

### Quantitative analyses

For the basilar artery, the mean SNR_BA_ for the DLR images was 46.2 ± 23.7, and it was significantly higher than that for the non-DLR images (SNR_BA_ = 28.5 ± 13.2) (*p* < 0.001). The CNR_BA_ for the DLR images (42.1 ± 22.3) was also significantly higher than that for the non-DLR images (CNR_BA_ = 25.7 ± 12.5) (*p* < 0.001). The SNR_BA_ and CNR_BA_ for the DLR images were 1.64 ± 0.38 and 1.66 ± 0.39, respectively, times higher than those for the non-DLR images.

The mean SI_BA_ in DLR and non-DLR images were 13,590 ± 781 and 13,515 ± 795, respectively. The mean SI_CSF_ in DLR and non-DLR images were 1248 ± 431 and 1401 ± 487, respectively. The difference of SI_CSF_ between DLR and non-DLR images (153 ± 131) was significantly larger than that of SI_BA_ between DLR and non-DLR images (74 ± 77) (*p* = 0.001).

The FWHM of the basilar artery in DLR and non-DLR images were 3.23 ± 0.67 mm and 3.25 ± 0.66 mm, respectively, and there was a significant difference between them (*p* = 0.006).

### Qualitative analyses

**Fig. 1:**
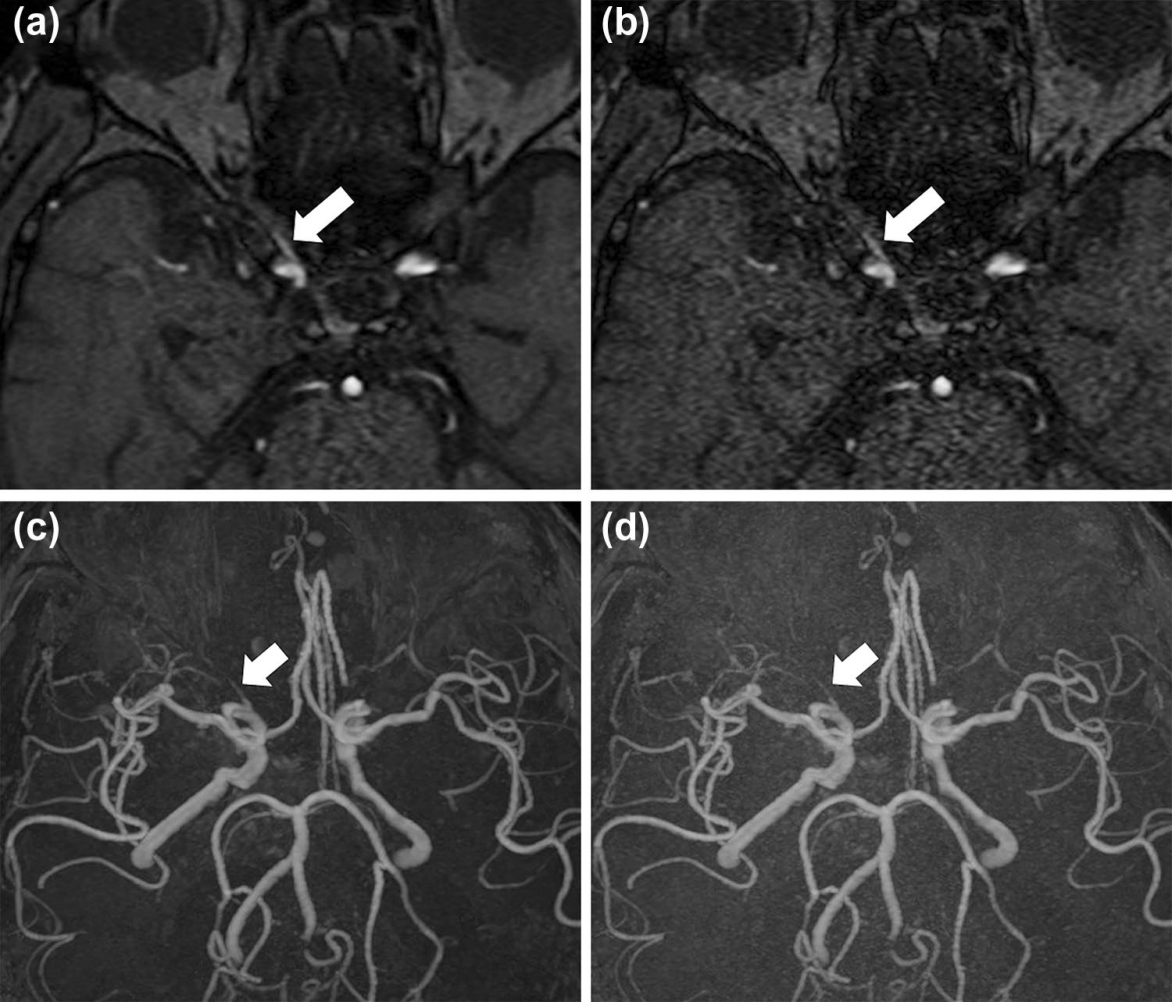
Depiction of the right ophthalmic artery (white arrows in **a**–**d**) in a 77-year-old man in MRA source (**a**, **b**) and MIP (**c**, **d**) images. In this patient, the depiction of the right ophthalmic artery in the DLR image (**a**, **c**) and non-DLR image (**b**, **d**) was rated as 5/4 and 4/2 by readers 1 and 2, respectively

**Fig. 2:**
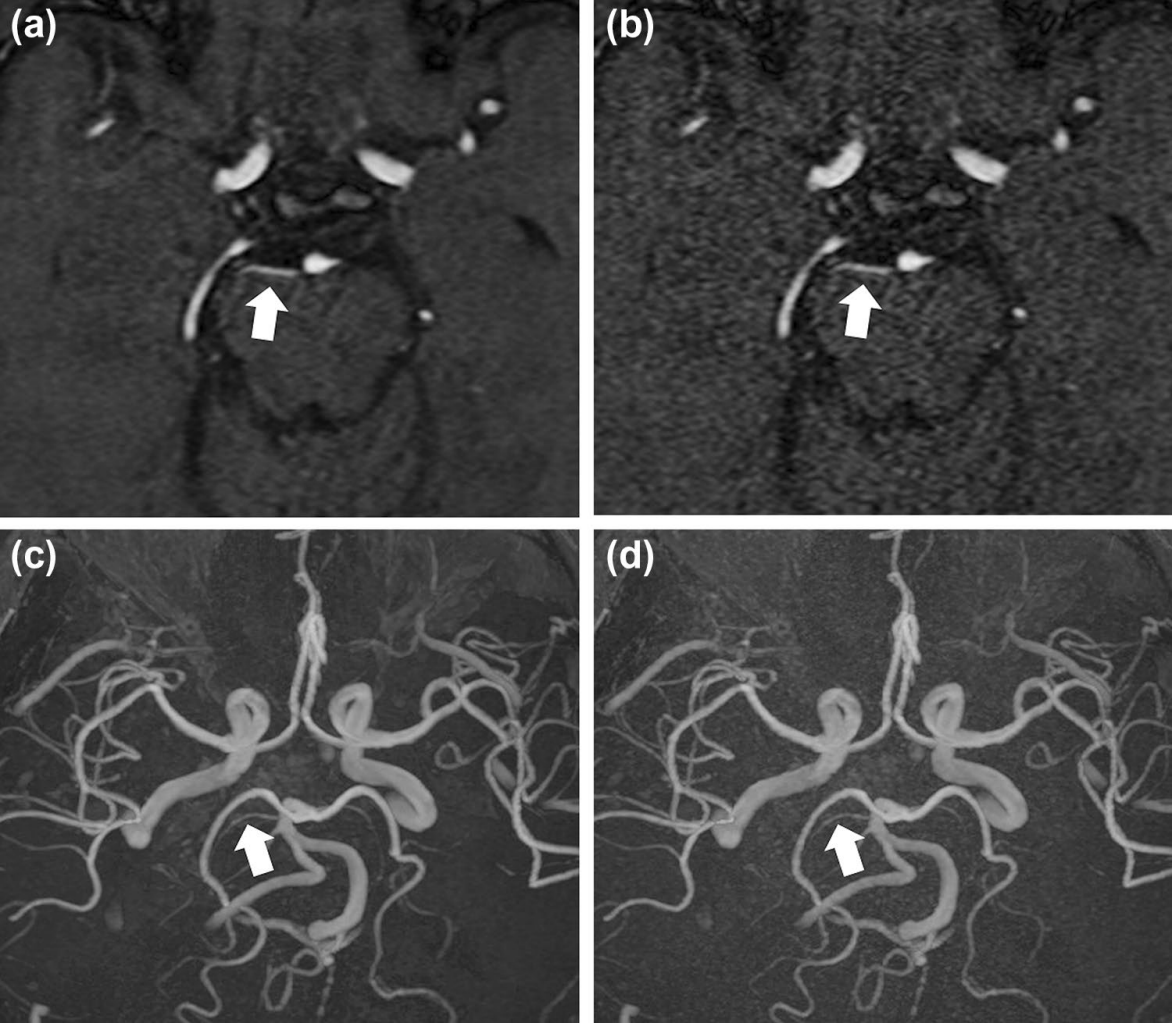
Depiction of the right superior cerebellar artery (white arrows in **a**–**d**) in a 61-year-old man in MRA source (**a**, **b**) and MIP (**c**, **d**) images. In this patient, the depiction of the right superior cerebellar artery in the DLR image (a and c)/non-DLR image (**b**, **d**) was rated as 5/4 and 4/3 by readers 1 and 2, respectively

**Fig. 3:**
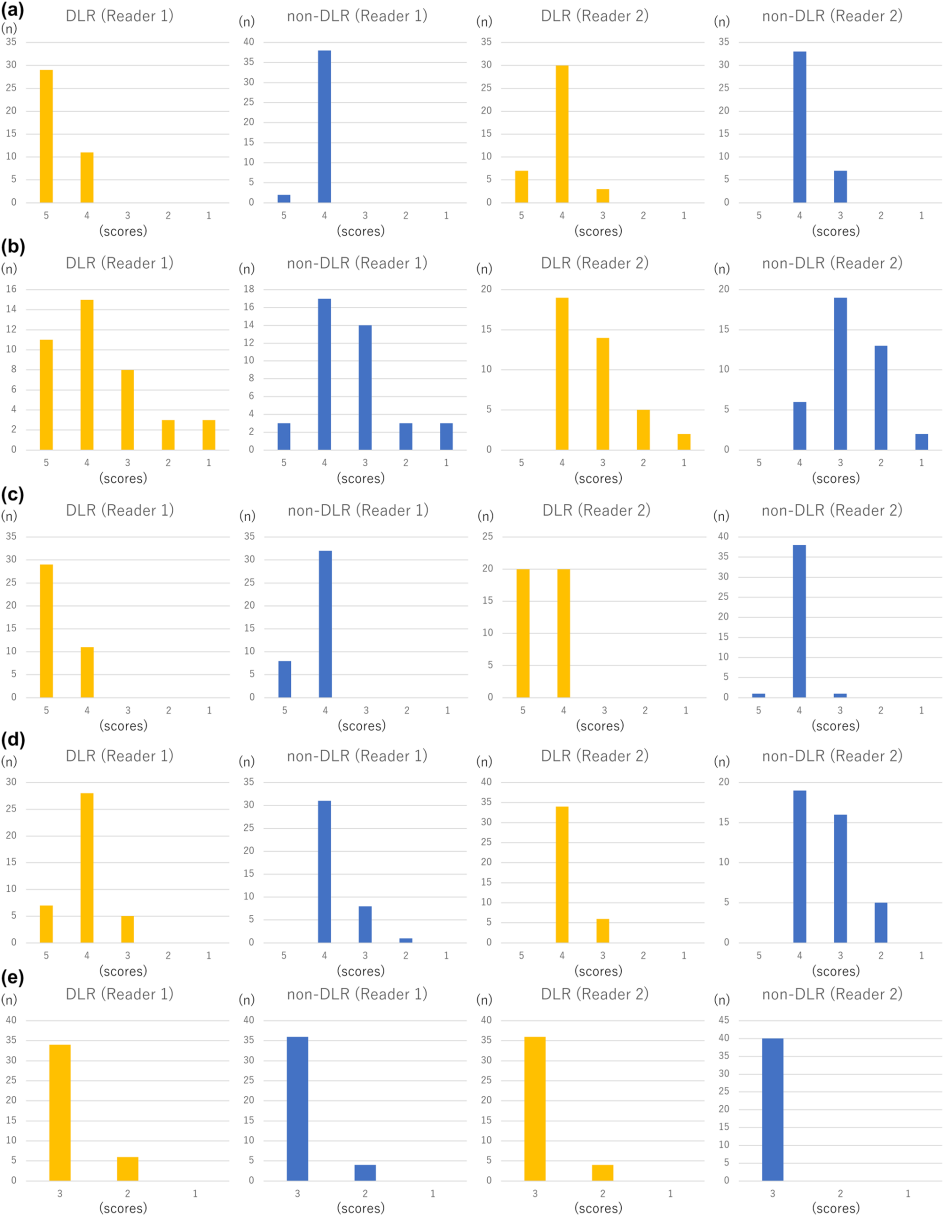

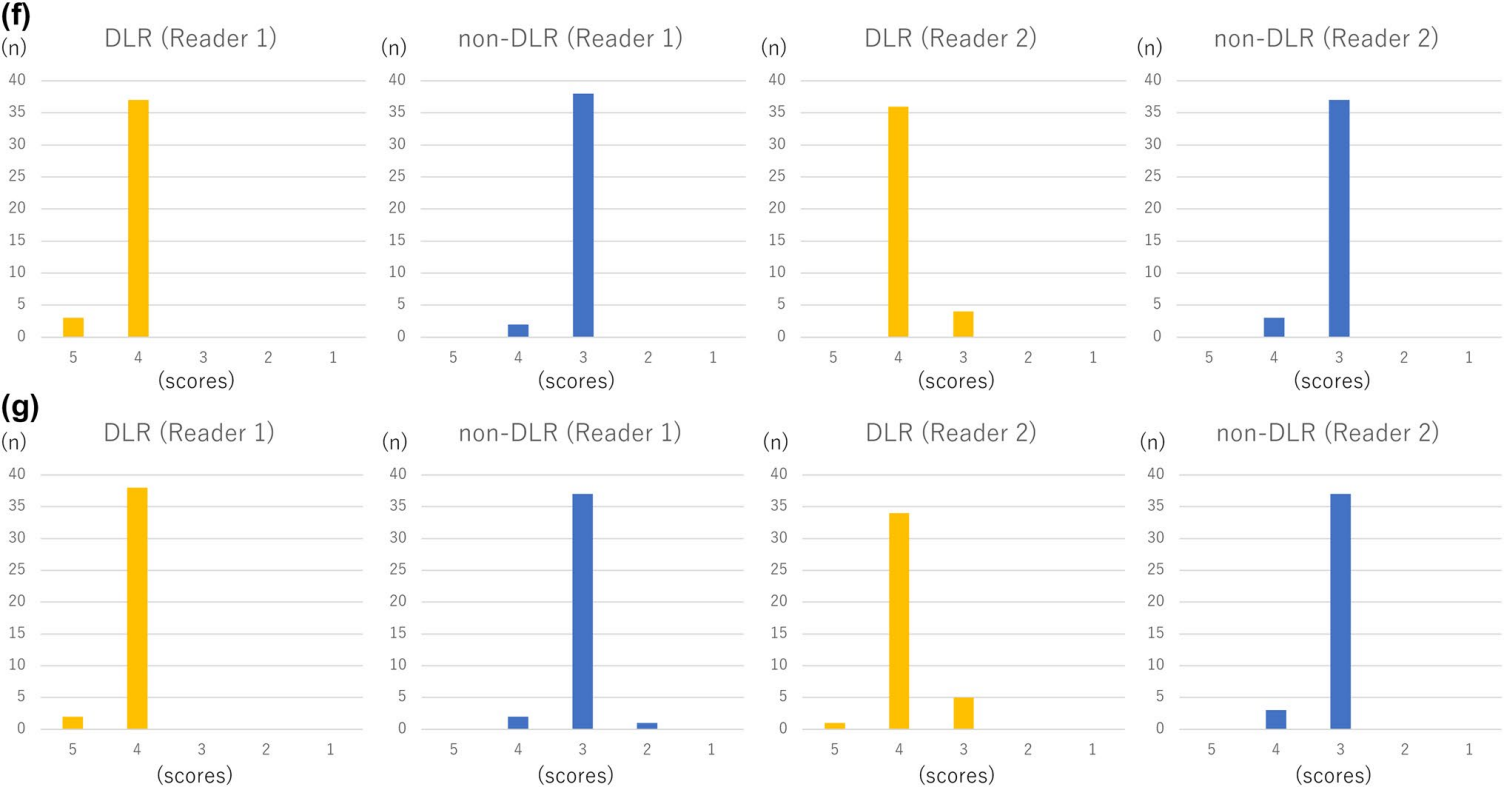
Results of subjective image analyses. The number of patients for each score in depiction of the internal carotid artery (**a**), ophthalmic artery (**b**), basilar artery (**c**), and superior cerebellar artery (**d**), artifacts (**e**), subjective noise (**f**), and overall image quality (**g**) is shown as bar graphs

According to both readers, the depiction of large arteries (internal carotid artery and basilar artery) (*p* < 0.001 and *p* < 0.005 for readers 1 and 2, respectively), depiction of small arteries (right ophthalmic artery and right superior cerebellar artery) (*p* < 0.003 and *p* < 0.003 for readers 1 and 2, respectively) (Figs. 1, 2), and subjective image noise (*p* < 0.001 for both readers) in the DLR images was significantly better than those in the non-DLR images (Table 1; Fig. 3). The overall image quality (*p* < 0.001 for both readers) of the DLR images was also better (Table 1; Fig. 3). However, there was no significant difference in terms of artifacts between the DLR and non-DLR images (*p* = 0.565 and 0.072 for readers 1 and 2, respectively).

**Table 1:** Results of subjective image analyses

	Reader 1			Reader 2			Interobserver agreement
	DLR	Non-DLR	*P* value	DLR	Non-DLR	*P* value	
Depiction of structures (5/4/3/2/1)							
Internal carotid artery	29/11/0/0/0	2/38/0/0/0	< 0.001*	7/30/3/0/0	0/33/7/0/0	0.005*	0.24
Ophthalmic artery	11/15/8/3/3	3/17/14/3/3	0.003*	0/19/14/5/2	0/6/19/13/2	0.003*	0.63
Basilar artery	29/11/0/0/0	8/32/0/0/0	< 0.001*	20/20/0/0/0	1/38/1/0/0	< 0.001*	0.56
Superior cerebellar artery	7/28/5/0/0	0/31/8/1/0	0.002*	0/34/6/0/0	0/19/16/5/0	< 0.001*	0.56
Presence of artifacts (3/2/1)	34/6/0	36/4/0	0.565	36/4/0	40/0/0	0.072	0.23
Subjective noise (5/4/3/2/1)	3/37/0/0/0	0/2/38/0/0	< 0.001*	0/36/4/0/0	0/3/37/0/0	< 0.001*	0.78
Overall image quality (5/4/3/2/1)	2/38/0/0/0	0/2/37/1/0	< 0.001*	1/34/5/0/0	0/3/37/0/0	< 0.001*	0.71

The number of patients for each score is shown. For interobserver agreement, kappa values are shown

*p < 0.05

In the analyses using Cohen’s weighted kappa, the interobserver agreement was fair to good for assessments of the depiction of structures (kappa value = 0.24–0.63), fair for assessments of artifacts (kappa value = 0.23), good for assessments of noise (kappa value = 0.78) and good for assessments of overall image quality (kappa value = 0.71).

## Discussion

DLR, which is available from MRI vendors, can reduce image noise. In this study, this technique successfully reduced image noise and provided clearer depictions of large and small intracranial arteries in MRA images obtained with a 1.5 T MRI unit.

MRI units with a magnetic field of 1.5 T are more readily accessible than 3 T units. However, MRA images obtained with 1.5 T units are associated with lower SNRs. This reduced SNR can lead to inferior depictions of small vessels [7, 8], remnants of coiled aneurysms [22], moyamoya vessels in patients with moyamoya disease [23] and feeders and drainers in arteriovenous malformation [24] in 1.5 T MRA images compared with 3 T MRA images. DLR is a promising technique that can improve the SNR regardless of the organs subjected to MRI [18]. This technique has the potential to reduce the existing deficiencies of 1.5 T MRA images. In this study, considering that evaluations of both large and small arteries are required for SSS-TOAST classification [3], we evaluated the depiction of large arteries (internal carotid artery and basilar artery) and small arteries (ophthalmic artery and superior cerebellar artery) of both the anterior and posterior circulations in 1.5 T MRA images. We achieved significantly clearer depictions of both large and small arteries using the DLR technique.

The use of DLR reduced the noise in the MRA images. There currently exist several techniques to reduce noise in MRI images, including increasing the number of acquisitions. However, this technique results in increased imaging time. Besides, the improvement in SNR is theoretically proportional to the square root of the number of acquisitions. Thus, to obtain twofold higher quality images in terms of the SNR, the imaging time would have to increase fourfold. Another way to reduce image noise in MRI is to increase the static magnetic field. However, as mentioned above, MRI units with higher static magnetic fields are less accessible. DLR provided higher quality intracranial MRA images with a higher SNR without compromising imaging time or accessibility. In the architecture of DLR technique, discrete cosine transform convolution is adopted to divide the data into a zero-frequency component path and a path, which is composed of 22 feature conversion layers, with 48 high-frequency components for reducing image noise [18]. This enables denoising while maintaining the contrast of images. It has been reported that the SNR of MRA images from 3 T imaging was 1.8–2.3-fold that of 1.5 T images [7, 8, 25–27]. Our study shows that using DLR to improve the SNR was close to the use of 3 T MRI units.

The FWHM of the basilar artery in DLR images was significantly shorter than that in non-DLR images. In this study, the difference of SI_CSF_ between DLR and non-DLR images was significantly larger than that of SI_BA_ between DLR and non-DLR images. This might have lowered SI of pixels at the boundary of the basilar artery which had SI of half the maximal SI of them, resulting in the shorter FWHM in DLR images.

There are some limitations to this study. First, we did not evaluate the detectability of diseases because such evaluations would require the inclusion of an adequate number of patients with each neurovascular disease. However, because it is evident that the depiction of arteries in the brain was significantly improved through the use of the DLR algorithm, it is worth conducting further investigations. Second, we did not compare the quality of 1.5 T MRA images with that of 3 T MRA images because the subjects would have to undergo both 1.5 T and 3 T MRI examinations with a minimal interval. A comparison of previous reports showed that using DLR to improve SNR was close to the use of 3 T MRI units [7, 8, 25–27]. However, to confirm this, direct comparisons will be needed in future studies. In addition, the depiction of arteries and the diagnostic performance in terms of detecting cerebrovascular disease should also be compared between 3 T MRA images and 1.5 T MRA images processed using DLR in future studies. Finally, the interobserver agreement was fair for some evaluation items (depiction of internal carotid artery and artifacts). Relatively large diameter of internal carotid artery might have caused various depictions at the center and peripheral part. Presence of several types of artifacts might have been associated with the relatively lower interobserver agreement for the artifacts. However, the results by both readers regarding whether there is significant difference or not between the DLR and non-DLR images were consistent for all the evaluation items.

In conclusion, DLR produced MRA images of higher quality, with less image noise and clearer depictions of arteries. Our study warrants future investigations to evaluate the effect of the DLR algorithm on the diagnosis of neurovascular diseases using MRA images.
